# Efficacy and Immunogenicity of Recombinant Pichinde Virus-Vectored Turkey Arthritis Reovirus Subunit Vaccine

**DOI:** 10.3390/vaccines10040486

**Published:** 2022-03-22

**Authors:** Rahul Kumar, Robert E. Porter, Sunil K. Mor, Sagar M. Goyal

**Affiliations:** 1 Veterinary Diagnostic Laboratory, Veterinary Population Medicine Department, College of Veterinary Medicine, University of Minnesota, 1333 Gortner Ave, Saint Paul, MN 55108, USA; kumar509@umn.edu (R.K.); porte349@umn.edu (R.E.P.); kumars@umn.edu (S.K.M.); 2 Department of Veterinary Pathology, College of Veterinary Science and Animal Husbandry, Pandit Deen Dayal Upadhyaya Veterinary Science University and Cattle Research Institute, Mathura 281001, Uttar Pradesh, India

**Keywords:** Turkey arthritis reovirus, recombinant vaccine, live virus vaccine, subunit vaccine, serum neutralizing antibodies, histologic lesion scores

## Abstract

We created a recombinant live pichinde virus-vectored bivalent codon optimized subunit vaccine that expresses immunogenic Sigma C and Sigma B proteins of turkey arthritis reovirus. The vaccine virus could be transmitted horizontally immunizing the non-vaccinated pen mates. The vaccine was tested for efficacy against homologous (TARV SKM121) and heterologous (TARV O’Neil) virus challenge. Immunized poults produced serum neutralizing antibodies capable of neutralizing both viruses. The vaccinated and control birds showed similar body weights indicating no adverse effect on feed efficiency. Comparison of virus gene copy numbers in intestine and histologic lesion scores in tendons of vaccinated and non-vaccinated birds showed a decrease in the replication of challenge viruses in the intestine and tendons of vaccinated birds. These results indicate the potential usefulness of this vaccine.

## 1. Introduction

Turkey arthritis reovirus (TARV) causes lameness in turkeys, generally at 12–17 weeks of age. A reovirus was isolated from the gastrocnemius tendons of turkeys affected with arthritis/tenosynovitis in the 1980s [1,2], but this condition was not observed for nearly 25 years until it was reported by Mor et al. [3]. Thereafter, several authors reported TARV-associated outbreaks of lameness in market age turkeys [4,5]. These outbreaks result in substantial economic losses to turkey farmers in the form of increased culling, increased condemnation rates, poor feed efficiency, and low rates of weight gain [4,6]. The disease has been experimentally reproduced to confirm the involvement of reovirus and the infection has consistently been associated with uni- or bilateral lameness due to swelling of the hocks, periarticular fibrosis, tenosynovitis, occasional erosion of the articular cartilage on distal tibiotarsus, and rupture of the gastrocnemius or digital flexor tendon [3,6,7,8].

The TARV is a member of genus *Orthoreovirus* in the family *Reoviridae* containing double-stranded segmented RNA genome in double-shelled capsid. The 10 segments of viral genome are classified as L class (L1–L3), M class (M1–M3), and S class (S1–S4) based on their electrophoretic mobility [9]. Reoviral genome has 12 open reading frames (ORFs), which encode eight structural and four nonstructural proteins [10]. Sigma C (SC) protein translated by the third ORF of S1 segment is an outer capsid cell attachment protein [11,12,13]. It is the most divergent among reovirus proteins and is the main immunogenic surface protein containing type- and broad-specific neutralizing epitopes [12]. Sigma B (SB) protein encoded by S3 segment is a major component of the viral outer capsid and contains a group-specific neutralizing epitope [14]. The SC protein alone or in combination with SB protein has been used in formulating subunit vaccines against avian reovirus infections [15,16,17,18].

Since 2011, breeder turkeys in the U.S. have been vaccinated with autogenous killed virus for TARV, but commercial market turkeys have not been vaccinated. Setbacks to a successful vaccination program for TARVs are the development of multiple variant TARV strains and the absence of a commercial vaccine. Recently, custom made autogenous vaccines with prevalent pathogenic TARV strains are being used by the turkey industry to vaccinate breeder turkeys. Breeders are being vaccinated to check the suspected vertical transmission and to provide maternally derived antibodies to the progeny in the initial days of their life. However, these autogenous vaccines are poorly characterized and show variable efficacy, especially against the variant strains [6]. Antigenic and genetic variants different from the vaccine strains have been reported from progeny and unvaccinated breeder flocks in the U.S. [3,5,8,19,20,21,22]. Outbreaks of TARV-associated lameness continue to be reported, and affected turkeys are generally sent to diagnostic labs.

This study describes a live recombinant pichinde virus-vectored codon optimized bivalent turkey arthritis reovirus (rPICV-TARV) vaccine that was developed using the SC and SB protein coding genes of the TARV SKM121 strain [23]. This study was designed to assess the safety, efficacy, and immunogenicity of the rPICV-TARV vaccine against homologous and heterologous virus challenge in the vaccinated poults. Another objective was to characterize the transmissibility of the vaccine and immunization from vaccinated to the non-vaccinated pen mates.

## 2. Materials and Methods

### 2.1. Vaccine Preparation

Live recombinant pichinde virus created using the reverse genetics system was used to develop a rPICV-TARV vaccine expressing the SC and SB proteins of TARV as antigens [23,24]. Briefly, the SC and SB gene inserts were ligated in the MCS region of plasmids *pP18S2-MCS/NP* and *pP18S1-GPC/MCS*, respectively, using T4 DNA ligase (ThermoFisher Scientific, Waltham, MA, USA) followed by transformation of DH5α and selection using ampicillin antibiotic. The recombinant plasmids were isolated using plasmid midi prep kit (Sigma Aldrich, St. Louis, MO, USA), PCR confirmed for reovirus genes, and sequence confirmed for correct orientation and reading frame. The recombinant plasmids were used to transfect BSRT7-5 cells using Lipofectamine^TM^ 300 transfection reagent (ThermoFisher Scientific, Waltham, MA, USA). The rescued virus was then grown in BHK-21 cells, and the expression of reovirus genes by the recombinant PICV vaccine virus was verified by RT-PCR and immune-fluorescent assay.

### 2.2. Virus

Turkey reovirus strains TARV O’Neil (ON) and TARV SKM121 (SKM) were propagated and titrated in Japanese quail fibrosarcoma (QT-35) cells. The 50% tissue culture infective dose (TCID_50_) was calculated using the Reed and Muench method [25]. The rPICV-TARV vaccine virus was grown in BHK-21 cells and titrated in Vero cells as plaque forming units [24].

### 2.3. Experimental Design

Day-old turkey poults (n = 180) were procured from a non-vaccinated and reovirus infection free flock. Ten poults were euthanized on the day of arrival for collection of tissue samples followed by blood collection. Serum samples were tested for reovirus antibodies by ELISA, and meconium, intestine, and tendon samples were tested by real time RT-PCR (rRT-PCR) to ensure reovirus infection and vaccination free poults. The poults were randomly divided into eight groups, namely (1) negative control (NC), (2) vaccine control (VC), (3) vaccinated and challenged with TARV SKM121 (V-SKM), (4) sentinels (for vaccine) challenged with TARV SKM121 (Sen-SKM), (5) non-vaccinated and challenged with TARV SKM121 (SKM) (to serve as SKM challenge positive control), (6) vaccinated and challenged with TARV O’Neil (V-ON) strain, (7) sentinels (for vaccine) challenged with TARV O’Neil (Sen-ON), and (8) non-vaccinated and challenged with TARV O’Neil (ON) (to serve as ON challenge positive control). The eight groups of birds were housed separately in air-filtered isolators with ad libitum food and water supply. Birds displaying signs of severe illness were euthanized according to the Institutional Animal Care and Use Committee (IACUC) protocol and guidelines from Research Animal Resources (RAR) at the University of Minnesota.

The detailed experimental plan is shown in Table 1. Briefly, poults were vaccinated with a primary dose of rPICV-TARV vaccine (0.2 mL, 3 × 10^7^ PFU/mL) by oral route at 2 days of age (doa). In groups Sen-SKM and Sen-ON, 12 birds/group were wing banded and added as sentinels after 2 days of primary vaccination (4 doa). Poults were boosted intranasally (except the sentinels and NC) with 0.2 mL (3 × 10^7^ PFU/mL) of rPICV-TARV vaccine at 9 doa. On day 14, blood samples were collected from the non-vaccinated, vaccinated, and sentinel birds for serology. Birds in the respective groups (except those in NC and VC) were challenged orally with 0.2 mL (3.2 × 10^7^ TCID_50_/_mL_) of TARV SKM121 or TARV O’Neil viruses at 15 doa. Groups NC and VC were sham inoculated with 0.2 mL of virus free culture media (MEM). The birds were examined daily for any overt clinical signs or mortality. Birds displaying signs of severe illness were euthanized according to the IACUC and RAR guidelines. Five birds from each group were euthanized on 21, 28, and 35 doa. Body weight of the euthanized birds was noted before sample collection at 28 and 35 doa. At necropsy, gross lesions were noted followed by collection of intestines (ileocecum) and hock joint with gastrocnemius and digital flexor tendons for real time RT-PCR (rRT-PCR) and histopathology.

### 2.4. ELISA and Serum Neutralization Test (SNT)

Serum samples were tested for anti-TARV antibodies using a commercial ELISA available at the Veterinary Diagnostic Laboratory, University of Minnesota (UMVDL) (https://www.vdl.umn.edu/node/14381, 20 December 2021). The sera were also tested for serum neutralization antibodies against virus strains TARV SKM12 and TARV O’Neil to characterize the homologous and heterologous virus-neutralizing capability of antibodies produced by the vaccinated and sentinel birds at Aviserve, Inc., Delaware. Briefly, the heat inactivated serum samples were 4-fold serially diluted in a 96-well plate, and 25 µL of reovirus preparations (100 TCID_50_) were added to all wells except negative control wells. Subsequently, the virus-sera mixture was incubated at 37 °C for 1 hour before adding onto freshly seeded primary hepatocellular carcinoma epithelial cells from a male leghorn chicken (LMH) (5 × 10^5^ cells/well) with 10% fetal calf serum and incubated at 37 °C for 4–5 days. Virus infected and uninfected cells were used as positive and negative controls, respectively. Virus controls, cell controls, and serum controls were included on each plate. The plates were observed daily for the appearance of reovirus specific cytopathic effects (CPE), e.g., cell swelling, syncytia formation, detachment from monolayer. Medium was removed on appearance of CPE and the cells were stained with a 1% crystal violet prepared in 10% buffered formalin for 2–3 min, followed by washing with warm tap water. Plates were air dried and antibody titers (cut off titer is 64) were recorded as the reciprocal of the highest dilution of serum that inhibited virus induced CPE in at least 50% of the cell monolayer.

### 2.5. Processing of Tissue Samples

Individual intestinal (ileocecum) samples (1 g) were homogenized in Hanks’ balanced salt solution (HBSS) for 1–2 min using a Stomacher (Model 80, Seward, Ltd., Worthing, West Sussex, UK) to prepare a 10% suspension. Individual tendon samples (1 g) were homogenized in phosphate buffered saline (PBS) for 2 cycles of 4 min each in Geno/Grinder tubes using Geno/Grinder (SPEX Sample Prep 2010 Geno/Grinder^®^, Thomas Scientific, Swedesboro, NJ, USA). Tissue homogenates were centrifuged at 1800× *g* for 10 min at 4 °C. The supernatant was decanted and frozen at −80 °C until tested by rRT-PCR.

### 2.6. Viral Nucleic Acid Extraction

Viral RNA was extracted from 50 μL of each intestinal and tendon sample homogenate using MagMAX™ Pathogen RNA/DNA Kit (Thermo Fisher Scientific, Waltham, MA, USA) on a Kingfisher-Flex instrument (Thermo Fisher Scientific, Waltham, MA, USA) following the manufacturer’s instructions. Extracted RNA was eluted in 90 μL of elution buffer.

### 2.7. Virus Gene Copy Number

Virus gene copy numbers in intestine and tendon samples were estimated using a universal avian reovirus rRT-PCR available at the Veterinary Diagnostic Laboratory, University of Minnesota (https://www.vdl.umn.edu/node/15341, 20 December 2021) using AgPath-ID™ (Thermo Fisher Scientific, Waltham, MA, USA). The One-Step RT-PCR kit (ThermoFisher, Waltham, MA, USA) was used following the manufacturer’s instructions. Briefly, 25 µL of reaction mix contained 12.5 µL of AgPath master mix, 1 µL of enzyme mix, 1 µL (10 µm/µL) of each primer, 1 µL (5 µm/µL) of each probe, 5 µL each of viral RNA, and nuclease free water. The reaction conditions were 45 °C for 10 min, 95 °C for 10 min, and 40 cycles of 95 °C for 15 sec and 60 °C for 45 s. A standard curve was plotted using 10-fold serial dilutions of TARV-positive RNA included with each 96-well plate. The gene copy numbers were calculated in intestine and tendon samples collected at different time points and the data was subjected to appropriate statistical analysis.

### 2.8. Histopathology

Soft tissues were fixed in formalin, trimmed, processed, and stained with hematoxylin and eosin (H&E). Formalin-fixed hock joints were decalcified in EDTA prior to processing for histopathological examination. Lesions in the gastrocnemius tendons were scored using a previously described histologic lesion scoring system [7].

### 2.9. Statistical Analysis

To neutralize the skewness, variability, non-normal distribution of data and small sample size, natural log of virus gene copy numbers in intestine and tendons was taken. A non-parametric Kruskal Wallis test followed by pairwise Wilcoxon rank sum test with continuity correction and “Benjamini and Hochberg (BH)” as *p*-value adjustment method was used to test the significance of difference in serum neutralizing antibody titers, virus gene copy number (in intestine and tendons), and histologic lesion scores in gastrocnemius tendons. Statistical significance was determined at *p*-value < 0.05. Statistical analysis was undertaken using R [26] and figures were produced using the package ggplot2 [27].

## 3. Results

### 3.1. Clinical Disease and Gross Lesions

During the period of this study, no reovirus-specific clinical signs, mortality, or gross lesions were observed in vaccinated and/or virus challenged groups. At all euthanasia time points, no reovirus-specific gross lesions, or abnormalities were observed.

### 3.2. ELISA and Serum Neutralization Antibody Titers

**Figure 1:**
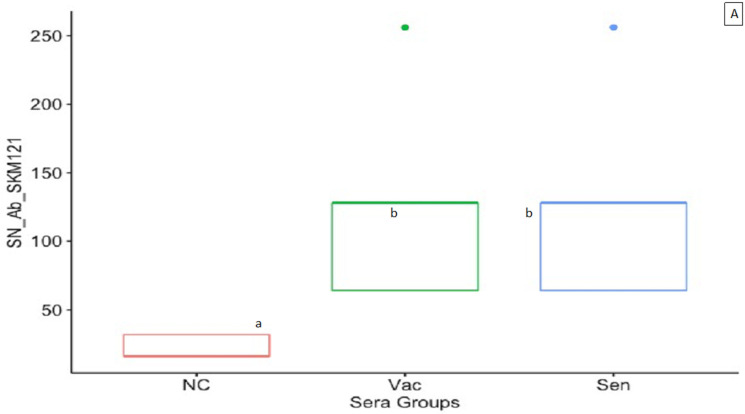

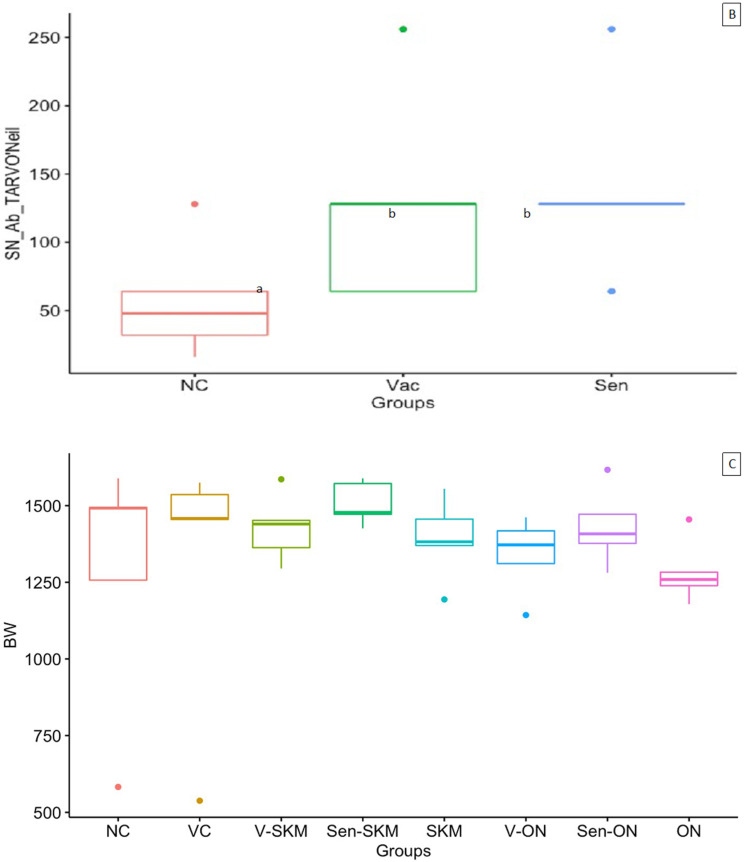

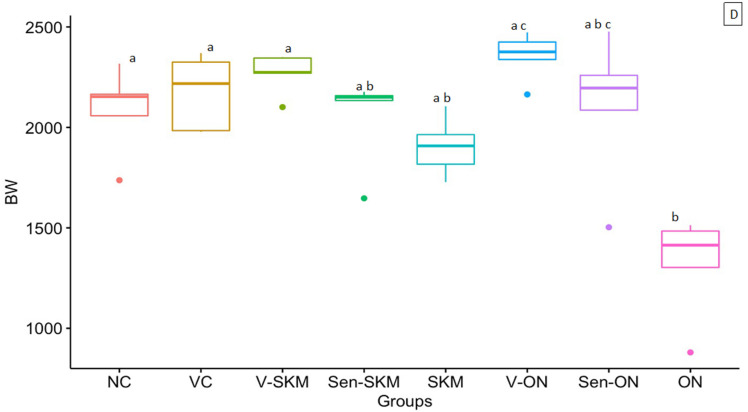
(**A**) SN Ab titer of the vaccinated and sentinel birds against the homologous TARV SKM121 virus; (**B**) SN Ab titer of the vaccinated and sentinel birds against TARV O’Neil virus; (**C**) BW of birds from respective groups at 28 doa; (**D**) BW of birds from respective groups at 35 doa. Box plots with different letters have significant differences at *p* < 0.05.

Sera from the vaccinated birds showed zero ELISA antibody titer and hence negative results, whereas based on our experience, the sera from experimentally infected birds usually show an ELISA antibody titer ranging from 740 to 1190 (unpublished data). Negligible to very low serum neutralization antibody titers against TARV O’Neil or TARV SKM121 were observed in sera from non-vaccinated negative control birds. The SN antibody titers against TARV O’Neil or TARV SKM121 in vaccinated and sentinel birds varied from 64 to 256 and were significantly higher than the negative control birds (Figure 1A,B).

### 3.3. Body Weight

At 28 doa (Figure 1C), the mean body weight of all the groups viz. NC, VC, V-SKM, Sen-SKM, SKM, V-ON, Sen-ON, and ON did not differ significantly, whereas the mean body weights of virus-challenge positive control groups (SKM and ON) were numerically lower than the respective vaccinated and sentinel groups (V-SKM, Sen-SKM and V-ON, Sen-ON, respectively). The mean body weight of the ON group was lower than all other groups. At 35 doa (Figure 1D), the mean body weight of the ON challenge positive control group was significantly lower than the mean body weight of NC, VC, V-SKM, Sen-SKM, and V-ON but did not differ significantly from Sen-ON and SKM positive control groups. The mean body weights of virus-challenge positive control groups (SKM and ON) were significantly lower than the NC and respective vaccinated groups (V-SKM and V-ON, respectively) whereas body weights did not differ significantly with the respective sentinel groups (Sen-SKM and Sen-ON, respectively). The mean body weight of the SKM group is significantly lower than V-SKM and V-ON groups. The mean body weight of the SKM group is significantly lower than the V-ON and Sen-ON groups and that of the ON group is significantly lower than the V-SKM and Sen-SKM groups. The mean body weight of the SKM group is numerically lower but did not differ significantly with the NC, VC, Sen-SKM, and Sen-ON groups.

### 3.4. Virus Gene Copy Number in Intestine

**Figure 2:**
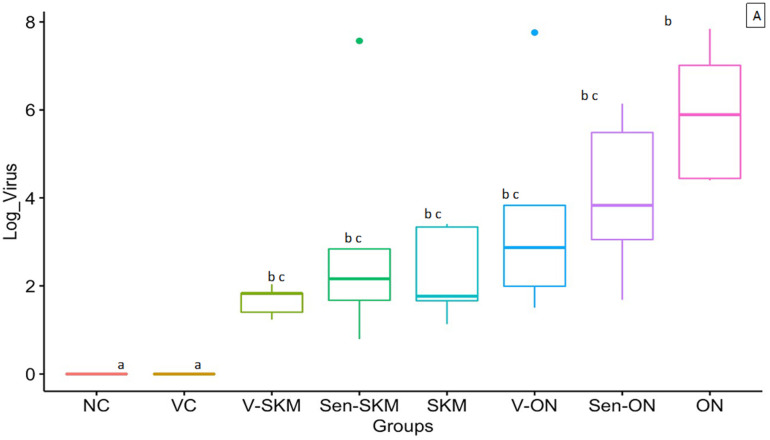

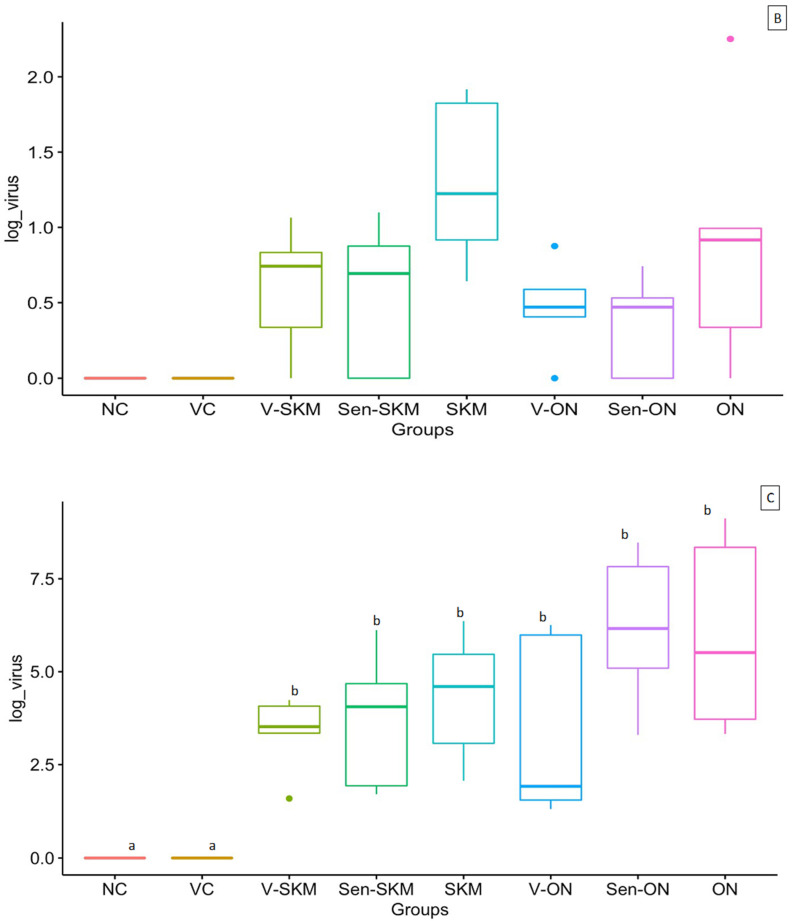

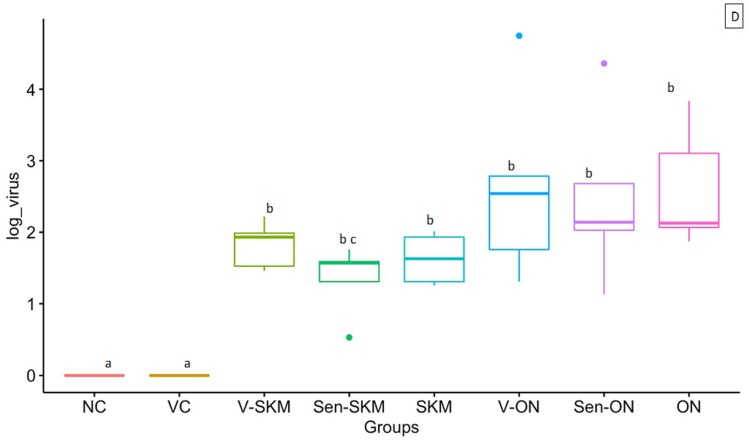
Virus gene copy numbers in birds from respective groups in (**A**) intestine at 21 doa; (**B**) intestine at 28 doa; (**C**) tendons at 21 doa; (**D**) tendons at 35 doa. Box plots with different letters have significant differences at *p* < 0.05.

At 21 doa (Figure 2A), virus gene copy numbers in NC and VC groups were significantly lower than the vaccinated-challenged (V-SKM and V-ON), sentinel-challenged (Sen-SKM and Sen-ON), and challenge-only (SKM and ON) groups. The highest virus gene copy numbers were observed in the ON group which are significantly higher than those of the NC, VC, V-SKM, and SKM groups. The virus gene copy numbers in the ON group were numerically higher but did not differ significantly with V-ON, Sen-ON, and Sen-SKM groups. The virus gene copy numbers in the SKM challenge-only group were numerically higher than the V-SKM and Sen-SKM groups, but those differences were not statistically significant. At 28 doa (Figure 2B), the virus gene copy numbers in all the groups did not differ significantly with each other. The virus gene copy numbers in the SKM and ON virus-challenge only groups were numerically higher than the respective vaccinated and sentinel challenge groups (V-SKM, Sen-SKM and V-ON, Sen-ON groups, respectively) but were not statistically significant. At 35 doa, sentinel groups challenged with SKM and ON viruses (Sen-SKM and Sen-ON) had numerically higher virus gene copy numbers than the rest of the study groups, but those differences were not statistically significant. The virus challenge-only groups (SKM and ON) had numerically higher virus gene copy numbers than the vaccinated groups but were not statistically significant.

### 3.5. Virus Gene Copy Number in Tendons

At 21 doa (Figure 2C), virus gene copy numbers in virus challenge-only groups (SKM and ON) were numerically higher than the respective vaccinated-challenge (V-SKM and V-ON) and sentinel-challenge (V-SKM and V-ON) groups but were not statistically significant. At 28 doa, vaccinated, sentinel, and virus challenge-only groups of SKM and ON viruses had numerically similar virus gene copy numbers. At 35 doa (Figure 2D), the virus gene copy number in the Sen-SKM group is significantly lower than the ON challenge-only group. Additionally, the virus gene copy number in the virus challenge-only groups (SKM and ON) were numerically higher than their respective vaccinated (V-SKM and V-ON) and sentinel (Sen-SKM and Sen-ON) groups but were not statistically significant. Virus gene copy numbers in NC and VC groups were significantly lower than the rest of the study groups at all time points (21, 28, and 35 doa).

### 3.6. Histopathologic Lesion Scores in Gastrocnemius Tendons

**Figure 3:**
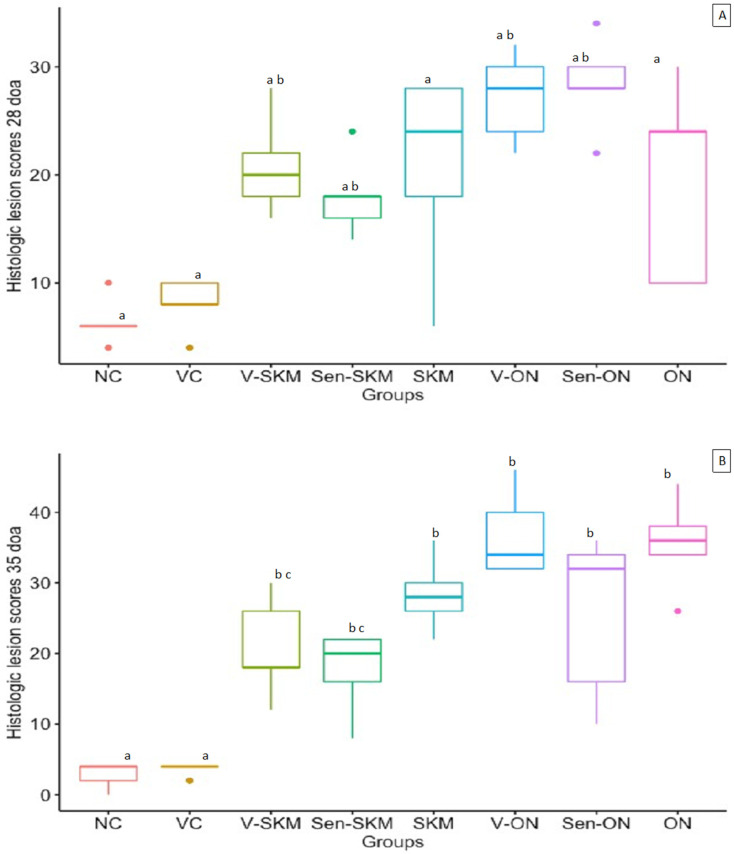
Histologic lesion scores in tendons of birds from respective groups at (**A**) 28 doa; (**B**) 35 doa. Box plots with different letters have significant differences at *p* < 0.05.

The histologic tendon lesions consisted of hypertrophy and hyperplasia of synoviocytes progressing to lymphoplasmacytic tenosynovitis and minimal fibroplasia at a later stage. At 21 doa, the mean tendon inflammation scores did not differ significantly in all groups. At 28 doa (Figure 3A), the histologic lesion scores in the NC and VC groups were significantly lower than the vaccinated (V-SKM and V-ON) and sentinel (Sen-SKM and Sen-ON) groups but did not differ significantly from the virus challenge-only groups (SKM and ON). At 35 doa (Figure 3B), NC and VC groups had significantly lower histologic lesion scores than the rest of the vaccinated, sentinel, and virus challenge-only groups. The mean tendon inflammation scores in the Sen-SKM groups were significantly lower than the SKM challenge-only groups in addition to V-ON and ON challenge-only groups. The mean tendon inflammation score in the V-SKM group was significantly lower than V-ON and ON challenge-only groups.

## 4. Discussion

In chickens, vaccination for ARV is primarily completed with live attenuated vaccine administered to young chicks followed by inactivated vaccine before egg laying [28]. This regime was found to induce the highest level of immune response in birds [29]. The absence of a commercial vaccine against turkey arthritis reovirus poses the biggest hurdle in the vaccination program. The turkey industry has adopted a strategy of using polyvalent autogenous vaccines in breeders prepared from the prevalent strains in their flocks [22,30]. Custom made autogenous vaccines are not the long-term solution because there are inherent drawbacks with autogenous vaccines discussed in detail elsewhere [18,22]. Emergence of variant reoviruses, especially variation in their cell attachment and outer capsid proteins is another hurdle in vaccination, because these variations cause inadequate protection provided by commercial vaccines in the vaccinated flock and their progeny. Our approach was to develop a live subunit vaccine to overcome these issues because subunit vaccines have potential advantages over the conventional and autogenous vaccines.

The live virus-vectored turkey arthritis reovirus vaccine provides an alternative to the use of autogenous vaccines which have the potential to promote the emergence of variant strains. The present study was designed to test the transmissibility and efficacy of the rPICV-TARV vaccine against homologous and heterologous virus challenge. The rPICV-TARV vaccine expressing SC and SB antigenic proteins has been shown to elicit a humoral immune response producing serum neutralizing antibodies in turkeys in our previous study [23]. Similarly, hemagglutinin and nucleoprotein genes of avian influenza virus (AIV) carried by rPICV vector to stimulate humoral and cell-mediated immune responses providing protection against pathogenic AIV in mice [31]. Previous studies have reported various subunit vaccines expressing SC and SB proteins against chicken and duck reoviruses [15,16,17,18]. Subunit vaccines against infectious bursal disease and adenovirus infection have previously been reported to be efficacious [31,32,33,34].

Most vaccine programs to protect young poultry are devised to either vaccinate breeders and passively immunize the offspring with maternal antibodies or to directly vaccinate the young birds with a live vaccine [35]. In our study, two-days old turkey poults were primed with 0.2 mL of rPICV-TARV vaccine (3 × 10^7^ PFU/mL) by oral route followed by booster dose (0.2 mL, 3 × 10^7^ PFU/mL) by intranasal route. Intranasal administration of a booster dose was undertaken to check the coarse spray administrability of our vaccine [23,28]. Poults were primed at two days and boosted at nine doa because birds are most susceptible to reovirus infection in their early life [35,36], and additional studies in our lab have shown that turkeys are susceptible to infection with TARV at least up to four weeks of age [37].

In our experience, turkey poults experimentally infected with TARV produce moderate to high antibody titers to whole chicken reovirus (commercial ELISA). In our study a similar commercial reovirus ELISA showed negative results with no antibody titers in vaccinated poults. This is likely because the ELISA used in the study uses whole virus rather than subunits as antigen; hence, the whole virus did not optimally present adequate epitopes to detect antibodies to the SC and SB proteins produced by the subunit vaccine. The ELISA would have been more sensitive for our purpose if subunits were used as the determinant antigen rather than whole virus [38]. The ELISA using SC and/or SB proteins targets for group and type-specific neutralizing antibodies as the coated antigen were reported to be better in predicting the level of neutralization antibodies than ELISA using the whole virus and showed a good correlation between ELISA and SNT [39,40]. Therefore, we intend to use subunit antigens in ELISA in our future studies. Poults vaccinated by a combination of oral prime and intranasal boost by the bivalent codon optimized rPICV-TARV vaccine produced serum neutralizing (SN) antibodies to homologous (TARV SKM121) and heterologous (TARV O’Neil) viruses. Both vaccinated and sentinel poults produced SN antibody titers (64–256) that were greater than that of control poults, suggesting the vaccine can be transmitted from vaccinated to non-vaccinated pen mates and induce the same level of immune response (Figure 1A, B). Based on our experience, the SN antibody titer of TARV-infected poults ranges from 32 to 512 (unpublished data). The subunit vaccine can likely produce a higher level of immunization and increase the SN antibody titers if the period between the prime and booster dose is increased to three weeks. The rPICV based vaccine needs at least three weeks to form memory cells in the immunized host for producing better immune reaction (personal communication with Drs. Ly and Liang). Similarly, kumar et al. recommended a gap of 2-3 weeks between the prime and booster dose [23]. The age at prime-boost (02 and 09 doa) and age at virus challenge (15 doa) was adopted in this study based on the previous research conducted on reovirus infection in chickens, with particular attention to the age susceptibility of chickens for reovirus infection. Chickens are most susceptible to reovirus infection during the first 14 days of their life and the birds become apparently resistant to reovirus infection at later ages [36,41,42,43,44,45]. Current unpublished work in our lab indicates that turkey poults are susceptible to infection with TARV for a much longer period, at least four weeks of age, which will allow us to modify the vaccine timing in our future studies [37].

By days 28 and 35 post vaccination, the vaccine control group (VC) birds showed no mortality and had mean body weights similar to sham-inoculated negative control (NC) birds, suggesting that the vaccine is safe and shows no adverse effects. Additionally, mean body weights of virus challenge-only groups (SKM and ON) were significantly lower than the respective vaccinated groups (V-SKM and V-ON, respectively) at 35 doa suggesting that the vaccine could check the replication of both the viruses in the intestine, thereby inhibiting the adverse effects of virus challenge translating into decreased body weight gain. The mean body weight of vaccinated groups (V-SKM and V-ON) was numerically higher than that of the respective sentinel groups (Sen-SKM and Sen-ON, respectively) but did not differ significantly. This decrease in the body weight gain of sentinel birds may be attributed to the higher population density in the sentinel groups.

At 21 and 28 doa, the virus gene copy numbers in the intestine and tendon of vaccinated-challenged (V-SKM and V-ON) and sentinel-challenged (Sen-SKM and Sen-ON) groups were numerically lower than the corresponding virus challenge-only groups (SKM and ON, respectively) suggesting that the subunit vaccine can inhibit virus replication in the intestine. These findings are likely attributable to the higher SN titers produced in the vaccinated and sentinel groups. Humoral immune response is the primary mechanism of providing protection against ARVs as antibodies produced against SC and SB proteins of ARVs inhibit virus attachment and cause lysis of virus and virus infected cells [22,46,47]. Additionally, the vaccine has provided similar protection against the homologous (TARV SKM121) and heterologous (TARV O’Neil) virus challenge. The vaccine was almost equally effective against heterologous virus challenge probably due to additional group-specific neutralizing antibodies induced by the SB protein of the vaccine. Precisely, SC protein elicits reovirus specific neutralizing antibodies and has been reported to elicit a strong mucosal immunity [48,49,50].

In all the vaccinated, sentinels, and virus challenge-only groups, the virus gene copy numbers in intestine and tendons were of the range of eight-fold log values at 21 doa, decreasing to two-fold log values at 28 doa in all the vaccinated, sentinels, and virus challenge-only groups. This decrease in the virus replication may be attributed to production of antiviral cytokines after 1 week of virus inoculation [50]. At 35 doa, the virus gene copy numbers slightly increased in both the sentinel-challenged (Sen-SKM and Sen-ON) groups. The probable reason behind this increase in virus replication is decreased level of circulating antibodies after 20 days of vaccination. Additional studies to optimize the vaccine dose and vaccination timing will likely increase the resultant antibody titers and extent of immunization.

Histologic lesion scores in V-SKM and Sen-SKM groups were lower than the SKM challenge-only group but V-ON, Sen-ON, and ON groups did not follow this pattern at 28 and 35 doa. This is probably due to increased levels of IL-6 and IFN-γ cytokines corresponding with lymphocytic infiltration in gastrocnemius tendon sheath, indicating the role of these cytokines in the development of tenosynovitis [51]. The joints and tendons act as sequestered sites; thus, preventing virus elimination by the immune system [40]. The increase in the histologic lesion scores of vaccinated (V-SKM and V-ON) and sentinel (Sen-SKM and Sen-ON) groups is less than the increase in the scores of virus challenge-only (SKM and ON) groups suggesting that the vaccine is possibly effective in controlling the tendon lesions.

## 5. Conclusions

The rPICV-TARV bivalent codon optimized vaccine has a potential to be used for vaccination against TARV infection. It can have promising economic benefits for the industry if breeder immunization is practiced preventing vertical transmission [52]. In birds, better immunity is achieved with a live vaccine before using inactivated vaccine in breeders [29]. We plan to study the immunogenicity of this vaccine in breeder turkeys in future work. Additionally, it is worth investigating whether our vaccine would interfere with other turkey vaccines. The dose, route, and number of shots will be further studied to develop an immunization program for turkeys for field use. The additional advantages of our recombinant vaccine is: (1) the characterized gene segments (SC and SB) of new variants can be easily cloned into the rPICV virus which will immunize the birds against the variant strains; (2) other genes of outer capsid proteins, e.g., M2 (MuB), can be cloned into the rPICV virus for enhancing the spectrum of our vaccine; (3) only gene segments translating to outer capsid proteins of variant TARVs are being used in the vaccine and not the whole viruses, eliminating the possibility of generation of variant TARVs. This study provides useful information that our vaccine could control and prevent the infection, thereby it paves a foundation for a promising potent future vaccine. Lastly, the importance of strict biosecurity and best management practices for maximizing vaccine efficacy in the prevention and control of reovirus infection in turkeys should not be ignored.

## Figures and Tables

**Table 1:** Summary of experimental design.

Group Name	Group Description (n)	Oral Vac (Days)	I/N Vac (Days)	Ch-SKM121 (Days)	Ch-ON (Days)	Sampling (Age)
**NC**	Neg ctrl (12 + 5)	-	-	-	-	21–28–35
**VC**	V-ONLY (12 + 5)	2	9	-	-	21–28–35
**V-SKM**	V-Ch-SKM121 (12 + 5)	2	9	15	-	21–28–35
**Sen-SKM**	V + Sent-Ch-SKM121 (10 + 12)	2	9	15		21–28–35
**SKM**	Ch- SKM121 (12 + 5)	-	-	15	-	21–28–35
**V-ON**	V-Ch-ON (12 + 5)	2	9	-	15	21–28–35
**Sen-ON**	Vac + Sent-Ch-ON (10 + 12)	2	9	-	15	21–28–35
**ON**	Ch-ON (12 + 5)	-	-	-	15	21–28–35

## Data Availability

Not applicable.
